# Differential Motor Facilitation During Action Observation in Followers and Leaders on Instagram

**DOI:** 10.3389/fnhum.2019.00067

**Published:** 2019-02-28

**Authors:** Sumeet Farwaha, Sukhvinder S. Obhi

**Affiliations:** Social Brain, Body and Action Lab, Department of Psychology, Neuroscience and Behaviour, McMaster University, Hamilton, ON, Canada

**Keywords:** social power, status, instagram, motor-evoked potentials, online status

## Abstract

High power and high socioeconomic status individuals have been found to exhibit less motor system activity during observation of another individual’s behavior. In the modern world, the use of online social networks for social interaction is increasing, and these social networks afford new forms of social status hierarchy. An important question is whether social status in an online setting affects social information processing in a way that resembles the known effects of real-world status on such processing. Using transcranial magnetic stimulation (TMS), we examined differences in motor cortical output during action observation between Instagram “leaders” and “followers.” Instagram Leaders were defined as individuals who have more followers than they are following, while Instagram Followers were defined as individuals who have fewer followers than they follow. We found that Followers exhibited increased Motor-evoked Potential (MEP) facilitation during action observation compared to Leaders. Correlational analyses also revealed a positive association between an individual’s Instagram follower/following ratio and their perceived sense of online status. Overall, the findings of this study provide some evidence in favor of the idea that our online sense of status and offline sense of status might be concordant in terms of their effect on motor cortical output during action observation.

**Statement of Significance:** This study highlights the importance of examining the effects of online status on motor cortical output during action observation, and more generally alludes to the importance of understanding online and offline status effects on social information processing.

## Introduction

Social interaction has traditionally taken place in a face-to-face setting between people who share the same physical space (Mathes, 1978; Baumeister and Leary, 1995). Within groups of people interacting in these settings, status hierarchies exist between individuals who are relatively high in status and those who are relatively low in status. As a result, there is a discrepancy between the associated thoughts and behaviors of those at various points within this hierarchy (Erber and Fiske, 1984; Graf et al., 2012; Maner and Menzel, 2013; Keltner and Cordaro, 2017). Numerous studies have examined the effects of status and social power on social cognition, perception and behavior (Fiske, 1993; Keltner et al., 2003; Smith and Trope, 2006; Guinote, 2007; van Kleef et al., 2008). It has been found that powerful individuals are significantly more goal oriented and devote less attention to less powerful others (Keltner et al., 2003) compared to individuals with a lower sense of social power (Ellyson and Dovidio, 1985; Fiske, 1993; Foerster et al., 2005; Fiske and Berdahl, 2007)^1^. Extant literature suggests that this dependence asymmetry between individuals of high and low status is linked with a host of effects on social information processing. However, very little is known about how social status in an online setting affects social information processing. One’s status can be regarded as a composition of their level of respect and esteem in society. Specifically, it refers to where an individual ranks relative to others in society (Adler et al., 2000). In contemporary society, it is vital to address the changing dynamic of social interaction. The rise of the internet has allowed individuals to partake in various forms of social interaction through the popular use of online social media. This new type of online interaction can take place in the form of instant messaging, commenting on a friend’s uploaded content, or “liking” the pictures they post on the social media platform. Given the prevalence of social media as a method of social interaction, it is important to examine whether online and real-world social interactions depend on the same cognitive processes, and whether the online world and the real world are concordant in terms of their effect on social information processing.

In today’s generation, individuals promote themselves and communicate with their peers primarily through the use of online social networks (Ridgway and Clayton, 2016). Currently, one of the most popular online social networks is Instagram (Sheldon and Bryant, 2016; Stapleton et al., 2017), which is used to share audio-visual content with “followers” (i.e., subscribers to their Instagram account). This social media application is often used on smart phones and provides users with several functions such as: (1) filtering their photos with the goal of attracting more likes, comments, and followers and (2) including keywords using hashtags (#), which relay the major themes of their post in the caption section (Lee et al., 2015). In this paper, we focus specifically on the nature of social interactions on Instagram, and examine the ratio between the number of “followers” an individual has versus the number of others that they are “following,” as a way to potentially index a form of Instagram status hierarchy. Extant literature suggests that those who have more followers than they are following on social media exhibit greater perceived online status and social power compared to those who have profiles with the opposite trend (De Souza and Ferris, 2015; McCain and Campbell, 2016; Sherman et al., 2016). Therefore, we ask whether individuals who have more followers than they are following are similar to high status power holders in the real-world. Specifically, we focus on the previously demonstrated effects of status and power on interpersonal sensitivity, where the observation of an action leads to the automatic activation of neural circuits in the observer, as if they were performing the action themselves (Oberman and Ramachandran, 2007; Iacoboni, 2009; Heyes, 2010; Obhi and Hogeveen, 2010; Rizzolatti and Sinigaglia, 2010; Hogeveen and Obhi, 2011). This motor cortical activity during action observation has been widely reported and is thought to be an important mechanism for processing other social agents (Iacoboni and Dapretto, 2006; Hari and Kujala, 2009). In the current experiment, we ask whether social status hierarchy on Instagram is associated with effects on interpersonal sensitivity that are similar to the known effects of real-world status and power.

Previous research has demonstrated a link between how powerful an individual feels and the degree of motor excitation they exhibit when observing another individual acting. Hogeveen et al. (2014) used transcranial magnetic stimulation (TMS) and electromyography (EMG) to examine whether such motor excitation associated with the observation of another person’s action would be lower in high-power relative to low-power individuals. They found that individuals primed to feel powerful showed a reduction in the amplitude of motor-evoked potentials (MEPs) during action observation compared to those primed to feel powerless (Hogeveen et al., 2014). Motor excitation is inferred in TMS studies from the amplitude of MEPs, which is recorded from the muscle of interest via EMG during action observation. To elicit an MEP, a single, fixed intensity TMS pulse is applied over an area of the motor cortex that corresponds to a muscle underlying the observed action (Hogeveen et al., 2014). Variations in the amplitude of MEPs correspond to changes in the excitability of motor cortical output (Fadiga et al., 1995; Fadiga et al., 2005). Thus, given that the degree of motor excitation during action observation is tantamount to interpersonal sensitivity (Petroni et al., 2010), these results suggest that powerful individuals may be less socially attuned to others, relative to individuals with a lower sense of power (Hogeveen et al., 2014).

In concordance with the TMS study of Hogeveen et al. (2014), recently, work showed that individuals with low socio-economic status (SES) exhibited stronger electroencephalogram (EEG) Mu-suppression when viewing another individual’s hand gestures, compared to their high SES counterparts (Varnum et al., 2016). Since Mu-suppression has been proposed as an indirect measure of mirroring activity (i.e., sensorimotor activity during action observation), this result was taken to suggest that mirroring is greater in those who are lower in SES (Varnum et al., 2016). Thus, together with the results of Hogeveen et al. (2014), this result supports the idea that higher levels of status and power are associated with lower levels of motor cortical output during action observation, compared to lower levels of power and status.

In this paper, we suggest that an individual’s “follower to following” (f/f) ratio can be used as an index for online sense of status (and power). Specifically, individuals with an Instagram follower to following ratio of <1 (fewer users following them relative to the number of users that they follow) might be classified as Instagram “followers” and those with a ratio of >1 (more users following them relative to the number of users that they follow) may be classified as Instagram “leaders.” Based on previous research examining status and power in the real world, we hypothesize that Instagram “followers” primed with their own f/f ratio will display increased motor cortical activity during action observation compared to Instagram “leaders.” When taken together with the literature introduced earlier in this section, such a pattern would suggest that the follower/following (f/f) ratio indexes a form of Instagram status, and that this online status exerts effects on how these individuals process other people (i.e., their level of interpersonal sensitivity).

## Materials and Methods

### Participants

38 volunteers (9 males, 29 females; *M* = 18.34 years, *SD* = 1.59) participated in this study for course credit. The sample size was determined based on numerous peer-reviewed between-group MEP studies, that achieved statistical power of 80% (*d* = 1.19; Fourkas et al., 2008; Fitzgibbon et al., 2012; Hogeveen et al., 2014). Participants were recruited from McMaster University’s Psychology, Neuroscience and Behaviour Research Participation System. All participants were right-handed and had normal or corrected-to-normal vision. In addition, all participants were naïve with respect to the purpose of the experiment. Most importantly, all participants were screened for contra-indications to TMS prior to participation. The study was approved by the McMaster University Research Ethics Board (MREB) and participants provided informed consent before participation.

### Materials and Methods

SuperLab (Version 4.2; Cedrus Corporation, San Pedro, CA, United States) was used to program this TMS experiment, and the stimuli were displayed on a 20-in. (50.8-cm) LCD monitor. The Magstim Rapid2 system was used to carry out the TMS. In addition, a Biopac psychophysiological recording system was used to record EMG data. MEPs were measured with surface electrodes placed over the abductor pollicis brevis (APB) muscle of participants’ right hand. Similar to previous studies examining MEPs, the EMG signal was acquired with a 5,000-Hz sampling rate, amplified (to 5.0 mV), filtered (bandpass 10–500 Hz), and sent to a laptop computer for offline analysis (Hogeveen and Obhi, 2012). All inferential statistical analysis was performed with SPSS statistics.

**FIGURE 1:**
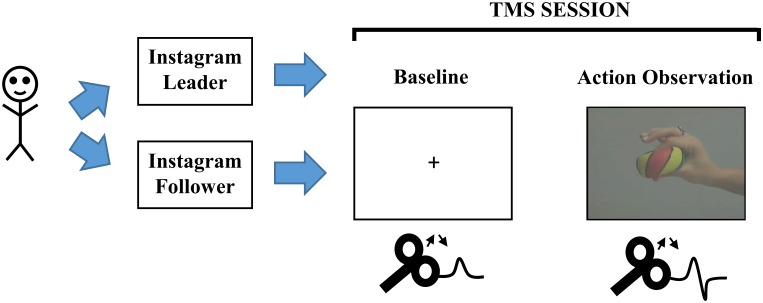
Schematic of the experiment. McMaster SONA, McMaster University Psychology Participant Pool; TMS, Transcranial Magnetic Stimulation.

The stimuli used in this experiment were clear videos depicting a right hand squeezing a rubber ball between the thumb and index finger (see Figure 1). The videos depicted the hand repeatedly squeezing the ball three to seven times.

The setup for TMS required the participant to first put on a swim cap, so that the experimenter could make markings for specific locations if necessary. The experimenter located the vertex and hand area of left primary motor cortex (M1) using a standard landmark technique (Hogeveen and Obhi, 2012). After the M1 area was found and highlighted using a washable marker, the experimenter used a coil holder and arm to ensure that the coil positioning was stable throughout the TMS experiment (Lepage et al., 2010). Participants were also asked to sit completely back on a chair and remain as still as possible throughout the experiment. The experimenter sat behind the participant to ensure that the Magstim coil positioning and participant was as stable as possible throughout the TMS experiment. Similar to previous studies in the literature, stimulator output was lowered to determine the minimum intensity capable of eliciting visible MEPs (∼1 mV peak to peak) on more than 50% of TMS pulses (Lepage et al., 2010; Enticott et al., 2012; Hogeveen and Obhi, 2012; Loporto et al., 2013). Stimulation intensity ranged from 45% to 71% (*M* = 58%) of stimulator output. During the first block of the TMS experiment, baseline motor cortical output was established by delivering 30 TMS pulses while participants viewed a fixation cross (75 total trials). As such, there were 30 trials with TMS stimulation and 45 trials without TMS stimulation. After the baseline block, participants began the action observation block in which each trial comprised a fixation cross for 2,000 ms, followed by videos of the hand squeezing action from 3,750 to 8,750 ms (75 total trials). During action observation blocks, 30 trials included TMS stimulation and 45 trials did not include TMS stimulation. TMS pulses were delivered at points of maximum squeeze intensity on 30 of the trials and occurred 3,128, 4,328, 5,494, or 6,728 ms after trial onset in both blocks (Hogeveen and Obhi, 2012). As a result, the task and temporal information during baseline and action observation were identical. Therefore, the only difference between both blocks was whether the participant saw a fixation cross or action video.

### Procedure

Participants completed the experiment in a testing room. They were seated in chair in front of a computer monitor before the TMS setup began. Once the researcher ensured that the coil was in a stable position overlying the left motor cortex of the participants, they were given instructions about the task. Specifically, participants were told that they were about to watch two separate sets of videos. During the first block of videos, they were asked to just focus on the fixation crosses that would appear in the middle of the screen one at a time. While they were focusing on these crosses, they were also asked to count the number of seconds each fixation was presented. During the second video, participants were asked to focus on the ball squeezing action. While they were focusing on this action, participants were also asked to count the number of squeezes contained in each video.

### Follower/Leader Priming

Importantly, before the participants were provided with any instructions about what they were about to watch on the computer monitor, they were asked to login to their Instagram account, write down how many followers they had and how many others they were following, and circle the larger number. In addition, they were also asked to indicate their perceived online status relative to their peers on Instagram by placing an ‘X’ on a 10 rung ladder, where those at the top had the most followers and those at the bottom had the least [adapted from Adler et al. (2000)]. These questionnaires effectively served to prime participants based on their f/f ratio and online status responses and also allowed us to classify them as Instagram leaders or followers. Before completing these questionnaires, participants were told that once they had completed their forms, they would need to place them into a file folder made available to them on their desk. The experimenter left the room while participants completed these forms and did not access this folder until after the experiment was complete. This procedure ensured that the experimenter was blind to the information provided by the participant and minimized any potential biases arising from the experimenter knowing the participant’s status as a “follower” or “leader.” After the TMS experiment, the information from these completed forms were used to categorize participants into an Instagram “leader” or “follower” group.

## Results

### Data Analysis

The dependent measure in the experiment was MEP facilitation, which refers to the percent change in MEP amplitude between the baseline block and the action observation block for each participant. The MEP signal was quantified using the peak to peak method, using Biopac’s Acknowledge software during offline analysis (Hogeveen et al., 2014). To examine differences associated with being a Leader or a Follower on Instagram, the sample was split into an Instagram Leader group and an Instagram Follower group. Participants were classified as Leaders if their followers/following ratio was >1, and as Followers if their followers/following ratio was <1. Finally, the data from all participants was used to examine the association between F/F ratio and Perceived Online Status, and the linear relationship between the F/F ratio and MEP Facilitation. Data was assessed for normality before conducting any statistical analysis.

### Range of F/F Ratios

Our sample included participants who had a wide range of f/f ratios, ranging from 0.16 to 1.95 (*M* = 1.01, *SD* = 0.55). In addition, perceptions of online status ranged from 1 to 9 rungs on the perceived online status ladder (*M* = 4.40, *SD* = 2.43).

### MEP Facilitation Analysis: Comparing Instagram Leaders and Followers

Prior to conducting inferential statistical analysis, trial data was examined for the presence of clear MEPs and trials were included or excluded accordingly. As a result, 10 participants had to be excluded from the analysis due to a lack of clear MEPs (fewer than 10) or excessive noise in the signal. For the included participants, we also removed specific trials per block based on the criteria of there being a clearly visible MEP. This resulted in the removal of 17.5% of trials in the baseline block and 13.9% of trials in the action observation block. Furthermore, for each participant, raw MEPs greater than 3 standard deviations from their mean were omitted from analysis (Hogeveen et al., 2014). This resulted in the removal of 1.03% data in the Baseline block and 0.57% in the Action Observation block. In regard to MEP Facilitation, participants with a mean change falling outside 2.5 standard deviations of the group average for each experimental condition (Leader, Follower) were excluded (Hogeveen et al., 2014). This procedure resulted in removal of one participant in the Follower group. After these pre-analysis procedures, the sample consisted of 13 participants in the Leader group and 14 participants in the Follower group.

**FIGURE 2:**
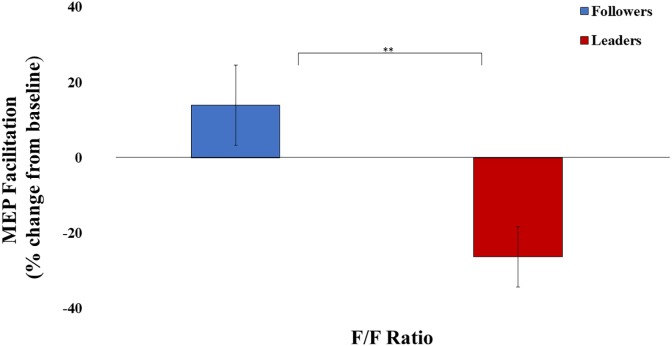
Motor-evoked potential (MEP) facilitation for both experimental conditions. ^∗^Significant at α = 0.05, ^∗∗^significant at α = 0.01, ^∗∗∗^significant at α = 0.001. Error bars indicate SEM.

The main question was examined by independent-samples *t*-test, with MEP Facilitation as the dependent variable and f/f ratio (i.e., leader or follower) as the independent variable. There was a significant difference between leaders and followers in MEP Facilitation, *t*(25) = 2.98, *p* < 0.01, *d* = 1.15. Specifically, Instagram followers (*M* = 13.9%, *SD* = 39.8%) displayed greater MEP facilitation compared to Instagram leaders, who appeared to show MEP suppression (*M* = -26.2%, *SD* = 28.8%) (See Figure 2).

In order to verify MEP facilitation and check whether MEPs changed over the course of a block, we divided the MEPs recorded during both baseline and experimental condition into two temporal bins (the first half of TMS trials within a block and the second half of TMS trials within a block), so that the data could be normalized within each temporal bin. For each bin, we performed a *t*-test comparing normalized MEPs against zero. The independent *t*-test against zero was significant for the follower group for both Bin 1 [*M* = 0.40, *SD* = 0.48, *t*(13) = 3.15, *p* < 0.01] and Bin 2 [*M* = 0.69, *SD* = 0.63, *t*(13) = 4.10, *p* < 0.01]. Thus, there was MEP facilitation during action observation for this particular group. However, we did not find a significant difference against zero for leaders for either Bin 1 [*M* = 0.05, *SD* = 0.73, *t*(12) = 0.261, *p* = 0.798] or Bin 2 [*M* = -0.07, *SD* = 0.28, *t*(12) = -0.872, *p* = 0.400]. Overall, there was motor facilitation for followers, but not for leaders. Finally, the fact that *t*-tests against zero were not different for bins 1 and 2 suggests similar MEP responses during early and late trials (i.e., that MEPs did not change appreciably over the course of a block).

### Correlation Between F/F Ratio and Perceived Online Status

The key question of whether an association existed between the f/f ratio and perceived online status was confirmed by a positive correlation *r* = 0.718, *n* = 27, *p* < 0.001.

### Linear Regression Between MEP Facilitation and F/F Ratio

**FIGURE 3:**
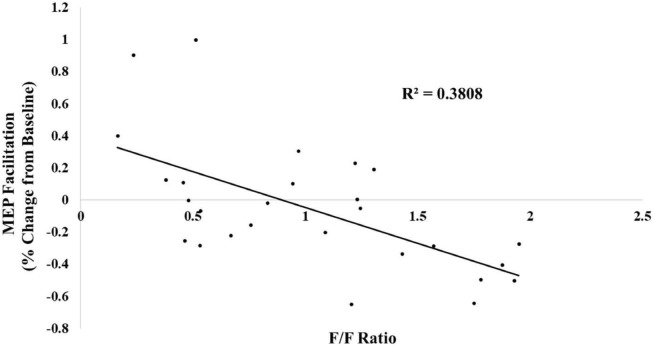
The following/follower ratio was linearly related with MEP facilitation.

For completeness, we treated the f/f ratio as a continuous variable and conducted a linear regression analysis to determine whether changes in the f/f ratio predicted changes in MEP facilitation. This regression was significant [β = -0.617, *t*(25) = -3.92, *p* < 0.01, *R*^2^ = 0.381] (see Figure 3).

## Discussion

The current study investigated whether priming individuals by asking them to provide their Instagram f/f ratio and to rate their own perceptions of online status, are related to MEP facilitation during action observation. Consistent with previous findings, our results showed that motor cortical activity was higher for observed actions in individuals primed with a low sense of online status compared to their high online status counterparts. More importantly, this study has extended previous research examining the influence of status and power on motor cortical output, by looking at an online index of social status, linked to user followers/following numbers on Instagram. We found that the Instagram follower group (individuals who had fewer followers than they were following; f/f ratio <1) exhibited an increase in MEP facilitation during action observation compared to the leader group, who appeared to show MEP suppression (f/f ratio >1). Regression analyses also showed that there was a strong negative relationship between the f/f ratio and MEP facilitation. In addition, there was a significant positive association between f/f ratio and perceived online status.

The amplitude of MEPs are an index of motor cortical output, which reflects the influence of the observed action on the motor system of the observer. The results of this study support previous findings from cognitive neuroscience studies showing that increases in both power and socioeconomic status are associated with decreasing levels of mirroring (Hogeveen et al., 2014; Varnum et al., 2016). The current study is the first (to our knowledge) to show that the sense of *online* status affects MEP facilitation. In other words, the sense of online status affects motor cortical activity such that those low in online status exhibit higher levels of activity compared to those high in online status.

The results of this study are consistent with previous claims that individuals of high status and power often fail to individuate others (Fiske, 1993; Russell and Fiske, 2010). That is, previous claims suggest that feeling powerful leads to less sensitivity to individuating information (and a correspondingly greater reliance on stereotypes) (Galinsky et al., 2003). In the present study, we find that feeling high in status reduces mirroring of observed actions – an effect that we suggest is tantamount to “reduced interpersonal sensitivity” (Buccino et al., 2004; Avenanti et al., 2010; Gutsell and Inzlicht, 2010). Although motor activation during action observation has been purported to relate to the capacity to process and comprehend the behavior of others, as well as important social capabilities such as empathizing and inferring mental states, direct evidence supporting some of these ideas is scarce (Agnew et al., 2007; Pfeifer et al., 2008; Lamm and Majdandžiæ, 2015). Despite this, the tendency for the brain to simulate (or “mirror”) the actions and experiences of others undoubtedly relates to sensitivity to the actions of others and has been reliably confirmed (e.g., Preston and de Waal, 2002; Rizzolatti and Craighero, 2004; Jackson et al., 2006; Lamm and Singer, 2010; Waytz and Mitchell, 2011; Bernhardt and Singer, 2012).

The current results are also in line with recent studies that have focused on examining the relationship between real life status and power and sensorimotor activity as indexed via measures such as EEG Mu-suppression and motor activity as indexed by MEPs elicited via TMS. Specifically, Varnum et al. (2016) have shown that lower socioeconomic status is associated with stronger Mu-suppression when viewing another’s hand gestures, suggesting that the putative human mirror system (HMS) may be more responsive among those who are lower in status. Similarly, Hogeveen et al. (2014) used TMS to elicit MEPs in individuals who were primed to a high power condition, a low power condition, or a neutral condition. Their results revealed a reduction in MEP facilitation in high power participants relative to low power participants (Hogeveen et al., 2014). Our study extends these results to perceptions of online status, and corroborates that high status seems to reduce the tendency to automatically mirror others. Given that differences in motor resonance have been linked to differences in status and power (Hogeveen and Obhi, 2013; Hogeveen et al., 2014; Varnum et al., 2016), it is surprising that very few studies have begun to address the question of whether a person’s online sense of status and offline sense of status are concordant or dissimilar in terms of their effect on motor cortical output. An important question for future work is when and whether online status and offline status exert similar effects (within participants) on a host of social cognitive processes beyond MEP facilitation during action observation.

Our results indicate that online sense of status and power is associated with differences in motor cortical output during action observation. These findings not only support previous studies examining the effect of real-life status and power on motor resonance, they also extend these findings to an online context. However, there are a number of potential limitations to our study. First, there was a gender imbalance between Instagram groups, there were more female participants in the Instagram Leader group (*n* = 12) compared to the Instagram Follower group (*n* = 9). Although this is not ideal, to our knowledge, gender differences in MEP facilitation during action observation have not been reported in the literature (Hari, 2006; Lepage et al., 2008). Second, the lack of a control muscle makes it problematic to extend this discussion to differences in *motor resonance* specifically between Instagram leaders and followers. This is because a strict definition of motor resonance requires the demonstration of muscle specificity, and because we did not record from a control muscle, we must limit our discussion to effects of status on motor cortical output during action observation. As such, future studies are encouraged to address this issue by including a control muscle unrelated to the action in question. Third, participants in this study engaged in only one baseline block before the action observation block, similar to previous studies examining group differences in MEP facilitation during action observation (Obhi et al., 2011; Hogeveen et al., 2014). A better approach would be to incorporate pre and post baseline blocks to take into account potential drifts/changes in motor cortical excitability across the experiment. This approach would allow a more definitive interpretation of any findings. As such, future studies are encouraged to adopt a pre and post baseline approach. Fourth, although we attempted to examine differences in *online status*, we did not collect information from participants about their actual *real-life status*. This leaves open the possibility that our results were driven by differences in real-life status rather than online status or that real-life status and online status are the same thing. While we acknowledge this possibility, we propose that understanding the relationship between real-life status and online status is critical for future work in that it relates to the potential attributes of different versions of the self (and there are cases in which these selves may be discordant). Despite this limitation, our data are consistent with our hypotheses and with previous studies from multiple labs. In addition, although the findings of this paper along with those from other recent papers (Hogeveen et al., 2014; Varnum et al., 2016) suggest that the link between power, status and mirroring is robust, we are unable to say anything about the precise functional role that “neural mirroring” might play in complex processes such as empathy and capacities such as decoding the actions of others. Even without this knowledge though, we suggest that automatic mirroring of others, can itself be used as a useful marker of sensitivity to the behavior of others (i.e., as a measure of “interpersonal sensitivity”).

In summary, we found that lower Instagram status is associated with higher levels of motor cortical output as indexed by MEP facilitation during action observation. These findings suggest that online and real-life status and power might exert concordant effects on motor cortical output. This pattern of data could account for the everyday experience that people in positions of power and those with high status sometimes seem less attuned to others compared to people who feel relatively low in status and power. Our work also opens up a new question about the effects of online status versus real life status on a host of other social cognitive processes. In this regard, future work should consider probing *the conditions in which* online and “real-world” status exert similar or different effects on social cognitive processing.

## Author Contributions

SF was involved in the recruitment, subject testing, data analyses, and writing up this report. SO was involved in the study design, data analyses, and writing up this report.

## Conflict of Interest Statement

The authors declare that the research was conducted in the absence of any commercial or financial relationships that could be construed as a potential conflict of interest.

## Footnotes

1 Status relates to power in that it often allows one to control the social outcomes of value to others. However, even though status and power are often considered together, it is important to note that they are separate constructs and can exert dissociable effects in certain cases.
