# Corrigendum: Differential Motor Facilitation During Action Observation in Followers and Leaders on Instagram

**DOI:** 10.3389/fnhum.2019.00323

**Published:** 2019-09-20

**Authors:** Sumeet Farwaha, Sukhvinder S. Obhi

**Affiliations:** Social Brain, Body and Action Lab, Department of Psychology, Neuroscience and Behaviour, McMaster University, Hamilton, ON, Canada

**Keywords:** social power, status, instagram, motor-evoked potentials, online status

In the original article, there was a mistake in Figure 3 as published. An incorrect version of the graph was accidentally uploaded in which variables were plotted on the wrong axes. The corrected Figure 3 appears below.

**Figure 3:**
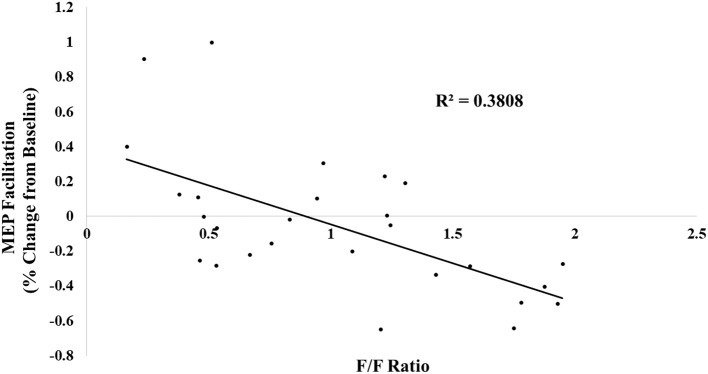
The following/follower ratio was linearly related with MEP facilitation.

The authors apologize for this error and state that this does not change the scientific conclusions of the article in any way. The original article has been updated.

